# Implementation of targeted screening for poverty in a large primary care team in Toronto, Canada: a feasibility study

**DOI:** 10.1186/s12875-021-01514-9

**Published:** 2021-09-30

**Authors:** Kimberly Wintemute, Meh Noor, Aashka Bhatt, Gary Bloch, Suja Arackal, Sumeet Kalia, Babak Aliarzadeh, Sabrina La Tona, Joyce Lo, Andrew D. Pinto, Michelle Greiver

**Affiliations:** 1 Department of Family and Community Medicine, North York General Hospital, 4001 Leslie street, LE140, M2K 1E1 Toronto, Ontario Canada; 2 Department of Family and Community Medicine, University of Toronto Practice-Based Research Network, Temerty Faculty of Medicine, University of Toronto, 500 University Avenue, M5G 1V7 Toronto, Ontario Canada; 3 North York Family Health Team, 240 Duncan Mill road, M3B 3S6 Toronto, Ontario Canada; 4 Department of Family and Community Medicine, St Michael’s Hospital, 36 Queen’s street East, M5B 1W8 Toronto, Ontario Canada; 5 Upstream Lab, MAP Centre for Urban Health Solutions, St. Michael’s Hospital, 36 Queen Street East, M5B 1W8 Toronto, Ontario Canada

**Keywords:** Family Practice, Primary Health Care, Mass Screening / organization and administration, Social Determinants of Health, Poverty, Poverty Areas, Feasibility Studies, Electronic Health Records

## Abstract

**Background:**

Poverty has a significant influence on health. Efforts to optimize income and reduce poverty could make a difference to the lives of patients and their families. Routine screening for poverty in primary care is an important first step but rarely occurs in Canada. We aimed to implement a targeted screening and referral process in a large, distributed primary care team in Toronto, Ontario, Canada. The main outcome was the proportion of targeted patients screened.

**Methods:**

This implementation evaluation was conducted with a large community-based primary care team in north Toronto. The primary care team serves relatively wealthy neighborhoods with pockets of poverty. Physicians were invited to participate. We implemented targeted screening by combining census information on neighborhood-level deprivation with postal codes in patient records. For physicians agreeing to participate, we added prompts to screen for poverty to the charts of adult patients living in the most deprived areas. Standardized electronic medical record templates recommended a referral to a team case worker for income optimization, for those patients screening positive. We recorded the number and percentages of participants at each stage, from screening to receiving advice on income optimization.

**Results:**

128 targeted patients with at least one visit (25%) were screened. The primary care team included 86 physicians distributed across 19 clinical locations. Thirty-four physicians (39%) participated. Their practices provided care for 27,290 patients aged 18 or older; 852 patients (3%) were found to be living in the most deprived neighborhoods. 509 (60%) had at least one office visit over the 6 months of follow up. 25 patients (20%) screened positive for poverty, and 13 (52%) were referred. Eight patients (62% of those referred) were ultimately seen by a caseworker for income optimization.

**Conclusions:**

We implemented a targeted poverty screening program combined with resources to optimize income for patients in a large, distributed community-based primary care team. Screening was feasible; however, only a small number of patients were linked to the intervention Further efforts to scale and spread screening and mitigation of poverty are warranted; these should include broadening the targeted population beyond those living in the most deprived areas.

**Supplementary Information:**

The online version contains supplementary material available at 10.1186/s12875-021-01514-9.

Contributions to literature

- Poverty affects health; screening and addressing poverty in primary care could lead to higher income and better lives
- This is rarely done in Canadian primary care
- We implemented a screening and income optimization program in a large “real world” community-based primary care team and evaluated its feasibility. Screening was targeted towards those living in the most deprived neighborhoods
- A quarter of patients living in deprived neighborhoods that visited the practices were screened and 20% of those screened positive, half of whom were referred for income optimization.
- Targeted screening may be a useful entry point towards universal screening; additional efforts to successfully implement targeted screening are warranted.

## Background

Socioeconomic status (SES) is one of the most important determinants of health [1]. Poverty adversely influences health outcomes for individuals [2–4]. Persons living with income insecurity have greater rates of chronic health conditions and a higher risk of reduced lifespan [3–5]. The COVID-19 pandemic has recently highlighted the effects of income disparities [6–8].

The pandemic has also highlighted gaps in socio-demographic data collection. Compared to international settings [7, 9], Canada lags in the collection and reporting of social determinants of health; these are associated with differences in COVID-19 infection rates and outcomes [10]. Some gaps have been partially addressed, leading to attention and efforts to direct appropriate resources to communities at greater risk [11, 12].

Primary care represents an ideal setting for the collection of information on social determinants of health, and for taking action on these [13–16]. Family physicians and their teams provide community-based longitudinal care for patients and families, building relationships based on trust, and generating knowledge about the context patients live in [13, 17].

In Canada, tax-funded insurance covers all medically necessary hospital and physician services for all citizens and permanent residents [18]. The pandemic has led to increased attention to social context, beyond biomedical issues. Family physicians have been provided with tools to address poverty and other social determinants of health during and after the crisis [19].

As with other major risk factors such as tobacco use, a critical first step involves clinicians asking their patients so that risk status can be identified and documented [20, 21]. An evidence-based tool for poverty screening and intervention in primary care has been developed and studied by Bloch et.al [22, 23]. It is central to current screening recommendations in Ontario and Canada [23].

However, in Canada, screening for poverty is not currently a routine part of family practice, with some exceptions [24]. Barriers include lack of provider training, lack of time, lack of knowledge and expertise, and difficulty changing workflows [25–28]. There may be multiple competing priorities as family physicians look after many issues and conditions that require attention. Pilot work in several sites in Toronto determined that poverty screening and intervention were feasible in the practices studied [14, 24]. However, these sites had a mandate to address social inequities as a priority, practice populations with high levels of poverty, dedicated champions and resources devoted to the intervention. An exploratory study in a variety of “real world” primary care settings found that only 9% of patients were screened, despite training and the presence of motivated healthcare providers [25].

New implementation strategies are needed to address currently low levels of poverty screening. We used Diffusion of Innovations theory to plan implementation [29]. According to theory, implementation may be more successful if the intervention is perceived as not being complex, as taking little time or practice resources, and as providing high value compared with usual care [29–31]. Our team had previous experience implementing screening interventions and integrating those in primary care Electronic Medical Records (EMRs) [32, 33]. We used this knowledge for the design of the poverty screen. We adapted approaches (EMR prompts, templates) found as part of other screening workflows that physicians were already using and were familiar with [34] to provide an intervention that was simple and easy to use.

An innovation in our approach was targeted screening for poverty. Using postal codes, we identified patients living in the most deprived neighborhoods. We expected that those areas included more patients living with poverty, and therefore would increase screening efficiency. The total number of patients requiring screening and the workload for physicians would be much lower than that required for universal screening. This would enhance trialability [29] through a limited initial implementation.

While universal screening for poverty is the recommended approach [16, 22, 23], targeted screening may present a useful initial step in the face of continuing limited update of screening. A similar approach (targeted vs universal screening) is currently being tested as part of a comparative effectiveness trial for major depression screening in adolescents, a screen with limited uptake [35].

If successful, further phases are being planned. A logic model is presented in Additional File 1. Phase One, reported here, consisted of assessing the feasibility of carrying out targeted screening for poverty and providing liaison to income security supports, and then assessing the feasibility of having a Case Worker collect income information sufficient to enable the calculation of sensitivity and specificity of the screening questions in this targeted study population. In Phase Two, we will determine sensitivity and specificity of the screening questions, when applied to a targeted population. In Phase 3, we will evaluate the effect of the intervention on patient household income.

### Objectives

Our primary objective was to evaluate the feasibility of targeted poverty screening in a large, community-based interprofessional primary care team. We also determined the feasibility of intervening to address poverty for those screening positive in a large, community-based interprofessional primary care team.

## Methods

### Study design

This was an implementation study with a progress-focused evaluation. We used the Standards for Reporting Implementation studies [36] to report the findings and the RE-AIM framework to report program elements [37].

### Setting

We implemented and evaluated the poverty screening strategy in a large community primary care inter-professional team in Toronto, Ontario, Canada, the North York Family Team (NYFHT, http://nyfht.com).

At the time of the project, NYFHT provided services at 19 clinical locations; the team included 86 family physicians practicing in office groups ranging from one to six physicians, and 40 Allied Health Providers. The NYFHT served 89,000 patients, most living in the north part of Toronto. The geographic area included neighborhoods with concentrations of high-income earners as well as pockets of poverty, including the provincial health region with the greatest absolute number and the second highest proportion of residents living below the low-income cut-off [38]. Prior to the study, most providers at the NYFHT did not routinely screen for poverty and there were no policies or formal educational efforts to address poverty.

NYFHT participates in the University of Toronto Practice Based Research Network, UTOPIAN [39]. UTOPIAN has expertise in the collection, management and linkage of EMR data.

### Participants

All 86 NYFHT physicians were invited to participate by email. We included patients of participating physicians that were 18 years of age or older as of April 1^st^ 2017, had at least one primary care visit over the three previous years, and were living in the most deprived neighborhoods.

External census information using the Statistics Canada postal code conversion file [40], which contains SES quintile data for small geographic areas (between 400 and 700 persons) by postal codes, was used to determine neighborhood-level deprivation [41, 42], and this data was linked with the NYFHT’s EMR data. We then proceeded to identify areas with quintiles indicating lowest income and greatest degree of material deprivation. In summary, material deprivation reflects neighborhood indicators for 1) proportion of people who have not graduated from high school, 2) ratio of employment to population, 3) proportion of adults living below the low-income cut-off [40]. A similar approach had been used in previous UTOPIAN projects [43, 44].

### Intervention

We devised an implementation strategy consistent with good design principles, considering inner and outer settings [31]. Briefly, a pilot was implemented and evaluated in a single office, with four motivated family physicians; this demonstrated initial feasibility in a community-based setting similar to ours. We scaled up using strategies known to be effective (presence of champion, building consensus on the importance of the issue, clinician education, leadership endorsement, availability of resources, adapting and tailoring strategies to local context) [31].

NYFHT’s Leadership endorsed the approach and provided resources, including a data manager and a project manager. The intervention lasted six months, from June 1^st^ 2017 to November 30^th^ 2017.

Interested physicians and their teams were offered a one-hour didactic education session prior to screening alerts being installed in their EMRs. During the session, clinicians were oriented to the importance of poverty screening; the clinical pathway for the study; and the availability of supports such as a case worker within the team. Case Workers assist people in finding resources in the community, including financial supports and housing. Following the education session, a referral to a case worker was added to the standard NYFHT referral form.

The medical records of adult patients with postal codes associated with the combination of lowest income and greatest degree of material deprivation were then flagged with an EMR alert. This prompted physicians or any other clinician accessing the chart to screen patients for poverty and load a standard template to record replies. To minimize cognitive load [45], the template also suggested standard workflows, including a referral to the case worker for positive screens (Additional file 2). The addition of the template to a record automatically turned off the alert.

Clinicians were prompted to ask the standard screening questions [2] during a visit for any reason. The screening questions are: “*Do you ever have difficulty making ends meet at the end of the month?*” (sensitivity 98%, specificity 40% for living below the poverty line) [2] and “*Have you filled out and sent in your tax forms?*”. Filling out the tax form can allow access to government benefits and a tax refund.

Patients with a positive screen had the opportunity to be referred to a trained FHT caseworker that assisted with income optimization and linkage to community support, at no cost to the patient. To optimize income, the caseworker provided information on available sources of income and subsidies; they then helped the patient fill out appropriate forms.

### Data and Process measures

We recorded the following process measures to assess feasibility of screening: proportion of physicians participating, number of charts flagged, number of patients screened, number of patients responding ‘yes’ to the first screening question, of those, number of patients with up-to-date income tax filing. We also assessed feasibility of the intervention to address poverty: number of patients referred to the case worker and number of patients who saw a caseworker. A summary of feasibility outcomes is provided in Table 1, differentiated by screening and intervening to address poverty for those screening positive.

**Table 1:** Feasibility outcomes according to the RE-AIM Framework

Reach	Number and percentage of all eligible patients with a visit over the six month period; number and percentage with a visit that were screened (screening)	509/852 (60%); 128/509 (25%)
Effectiveness	Number and percentage of patients screening positive; number having filed tax return (screening)	25/128 (20%); 25/25 (100%)
	Impact of the intervention on income; for this phase, we tested ability to collect income information through number and percentage of patients seen by the case worker with information available (intervention to address poverty)	8/8 (100%)
Adoption	Number and percentage of eligible physicians that participated in the program (screening)	34/86 (39%)
Implementation	Number of and percentage of charts labeled with alerts (screening)	852/27,290 (3%)
	Number and percentage of patients screening positive that were referred to the Case Worker (intervention to address poverty)	13/25 (52%)
	Number and percentage of patients that saw a case worker out of those referred; number and percentage out of all patients flagged (intervention to address poverty)	8/13 (62%); 8/852 (1%)

We recorded the number of physicians with a positive reply to the email invitation. UTOPIAN data was used to determine the number of patients in deprived neighborhoods. A data clerk entered the alerts in each chart.

We used EMR data for age and gender of patients; counts of patients screened; presence of the standard template in the chart (this indicated that the patient had been screened and that the screen was recorded); number of referrals and number of patients seen by the case worker. Data in the templates were used to determine answers to the screening questions. Following signed, informed consent, we surveyed patients seen by the case worker to record household income, number of financial dependents [22] and receipt of social benefits.

We used descriptive analyses (counts, proportions) for our results.

## Results

Thirty-four physicians (39% of the 86 team physicians) agreed to participate in the study, ten of whom (29%) were male; 29% of NYFHT physicians (25 / 86) were male. There were 27,290 patients aged 18 or older, with at least one encounter in the previous three years, in the practices of participating physicians. 852 patients (3%) were found to be living in the most deprived neighborhoods; an alert to screen for poverty was added to their electronic chart.

**Fig. 1:**
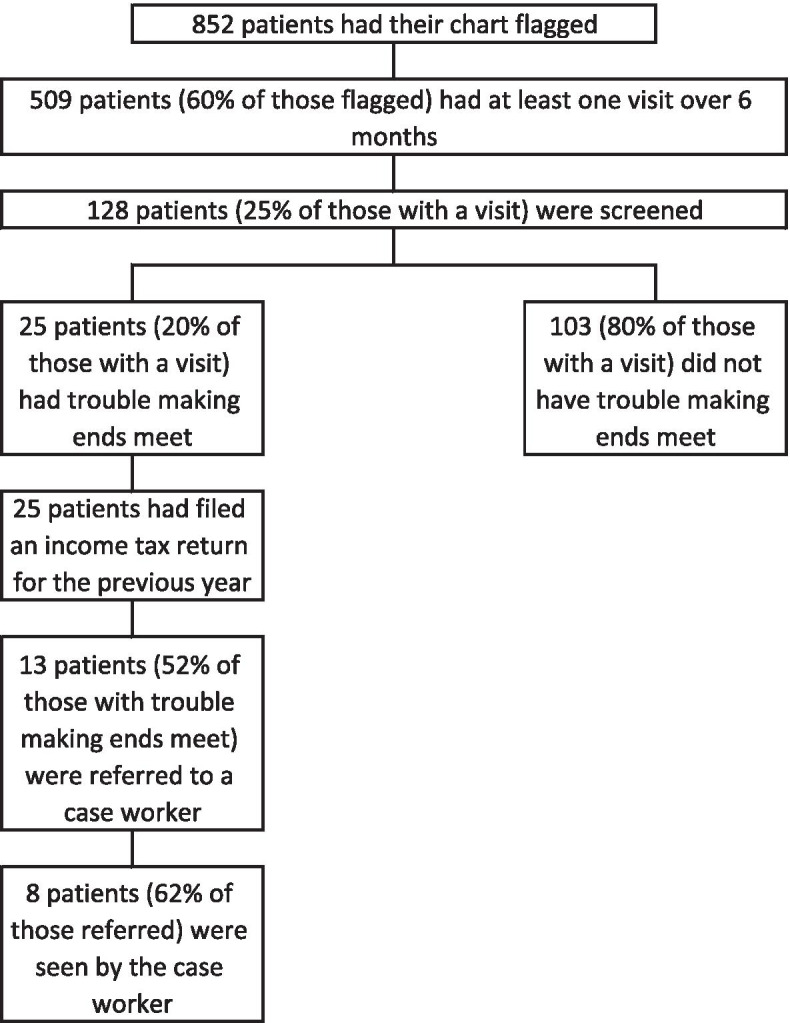
Flow diagram for patients flagged as living in deprived neighborhoods

Figure 1 provides a flow diagram and process measures for patients whose charts were flagged for poverty screening; Table 1 provides numerators and denominators for the different steps. There were 852 patients living in deprived areas; 509 (60%) had at least one visit over the 6 months of follow up; 128 patients (25%) of those with a visit had screening information (the standard template) entered in their record. 25 patients (20% of those screened) reported having trouble making ends meet, and all those had also filed their income tax reports for the previous year. 13 patients (52% of patients screening positive) were referred to a caseworker. Eight patients (62% of those referred) were seen by a caseworker during the study period. Patients seen by the caseworker represent less than 1% (8/852) of all flagged patients.

Table 2 provides patient characteristics by age range and sex. Ages ranged from 18 to 95 years old. Patient age ranges and sex were generally similar for those living or not living in deprived areas.

**Table 2:** Patient characteristics by age ranges and sex

		Patients not living in the most deprived areas, N (%)^a^	Patients living in the most deprived areas, N (%)^a^
TOTAL		26438	852
Age range (years)	18-24	3331 (13)	93 (11)
	25-34	4188 (16)	177 (21)
	35-44	4676 (18)	165 (19)
	45-54	4518 (17)	151 (18)
	55-64	4057 (15)	111 (13)
	65-74	2920 (11)	90 (11)
	75-95	2748 (10)	65 (8)
Sex	M	9557 (36)	305 (36)
	F	16881 (64)	547 (64)

^a^percentage may not add up to 100% due to rounding

Table 3 summarizes the characteristics of study patients seen by a caseworker. The mean annual income was $21,989.

**Table 3:** Characteristics of study patients who were seen by caseworker

Total number, N	Number female, N	Mean age (range)	Number already receiving social benefits, N	Mean annual income	Number with financial dependents, N
8	4	48 (29–63)	4	$21,989^a^	5

^a^Income was self-reported for seven patients and was confirmed through documentation for one patient

During the study period, 85 additional patients (whose charts had not been flagged) were seen by the case worker for income optimization.

## Discussion

Within the “routine” context of a large community-based primary care setting, we successfully identified people living in the most deprived neighborhoods and added clinical prompts to screen for poverty to their charts. A quarter of all patients flagged and with at least one visit were screened during a six-month period; 20% of those screened positive. A third of those patients were evaluated for income optimization by a caseworker, representing less than 1% of all patients flagged.

Our results demonstrate the challenges of implementing screening for poverty under usual circumstances in community-based primary care. We also demonstrate the feasibility of adding external census data to EMRs for clinical purposes, and of using targeted screening as a potentially useful method to initiate poverty screening for practice populations.

In our study, 25% of patients at risk were screened. This was in the context of limited health care provider training and multiple other priorities competing for clinician attention. This may be more representative of “routine” primary care in Canada than settings with a mandate to address poverty and other social determinants of health [14]. A similar study in a Community Health Centre, with such a mandate, found that 20% of patients were screened [14]. Although physicians welcomed screening tools, the authors noted that screening was likely not feasible if the physician was solely responsible, and recommended a team based approach [14]. A recent study of universal screening in “real world” primary care settings reported that 9% of patients had been screened [25]. Participating physicians were self-selected early adopters, committed to the process; 28% of patients screened positive, possibly indicating some targeting of patients. In our study, 39% of physicians participated, representing a less selected group (early majority) [29]. The EMR prompts could be seen by any clinician in the practice upon loading the chart; it is possible that a non-physician, such as a practice nurse, provided the screen, although we did not collect these data.

Following the RE-AIM framework [37], we report decreases at each step of the process. The largest decrease was at the initial, screening step. However, gaps developed at each following step and about 40% of patients referred did not go for an appointment. While we had taken steps to address known barriers for different stages, further exploration of specific barriers at each step, followed by specific strategies to address these are needed.

The proportion of the targeted population that received assistance with income optimization was small (1%). We do not know whether the assistance will result in increased income for those patients, whether the screening can be extended to those not living in deprived areas or could potentially become universal, or whether scaling up to other primary care settings, including non-interprofessional groups, is feasible. A large Canadian randomized controlled trial addressing several of these issues is currently under way [46].

In person primary care visits have plummeted during the Pandemic [47], limiting the ability to screen during these encounters. We do not know to what degree virtual care (phone, video visits) is associated with screening opportunities, nor what the mix of in person versus virtual visits will be, post-pandemic. It may be possible to screen for poverty outside of encounters; as an example, automated questionnaires can be securely emailed by primary care teams to their practice populations, with replies incorporated in EMRs [48].

Unexpectedly, we noted that the number of patients seen by the case worker for income optimization (85 patients) greatly outnumbered those seen through targeted screening (8 patients), possibly indicating knowledge gained or increased comfort with referrals to the case worker. Following the project, the two screening questions were added to the templates used by NYFHT physicians for routine periodic preventive health checks.

Societal inequities, including poverty, are a major public health concern. The goal should be to alleviate poverty for all those impacted, to improve their health and lives. While this intervention does not meet that goal, it provides information on a new pathway, through the identification of groups of patients at risk at the point of care. Calls for action to support those affected include social prescribing, enhanced preventive and chronic disease management visits in health care settings, and better access to psychological therapies [49]. A key point of entry is the identification of persons living with income insecurity; measures to enhance this include appropriately funding and supporting screening and making it a priority in healthcare settings.

### Strengths and limitations

A strength of this study is its context: it is situated within a large, multi-site community-based primary care team not specifically focused on social determinants of health. This improved the generalizability of our results.

Insights into real-world implementation challenges are highly valued by decision-makers [50]; this study provides information on these challenges and we were able to address several barriers to implementation. Physicians had reported not being able to remember to screen and not remembering what questions to ask [14]; we added alerts and templates that included the questions. Documentation in the EMR can be challenging [14]; standardized templates made this task easier. A lack of resources for those screening positive could pose challenges [14]; a referral to a NYFHT case worker was easily available for patients identified as screening positive and this was suggested in the template.

The study had several weaknesses. While a large substantial proportion of physicians volunteered, this was a self-selected convenience sample, and we did not address barriers or explore reasons for not participating. We were not able to quantify how many unreachable people were missed by our screening strategy because they do not access primary care clinics at all. We could not capture income information on all patients that were screened; income data reflects only the sample seen by the case worker which would preclude calculations of sensitivity and specificity of screening for the targeted population. NYFHT had resources, such as a case worker and data manager, that may not be available to non-interprofessional teams.

The study duration was six months; it is possible that a longer period may have resulted in more patients being referred to a caseworker. Neighbourhood-level SES does not necessarily reflect individual SES; our targeted approach will miss “hidden poverty”.

## Conclusions

A targeted poverty screening program combined with resources to optimize income for patients can be implemented in a large, distributed primary care team, although adoption was only partial. Efforts to address challenges to implementation, scale and spread screening for poverty, broaden the targeted population and optimize income for many more of those living in poverty, are warranted.

## Supplementary Information

**Additional file 1.** Logic model.

**Additional file 2.** Standardized template for poverty screening and workflow.

## Data Availability

Data generated during this study area included in the published article. Data from UTOPIAN to support findings are available from UTOPIAN https://www.dfcm.utoronto.ca/utopian-data-safe-haven, but restrictions apply to the availability of these data.

## Abbreviations

EMR: Electronic Medical Record
NYFHT: North York Family Health Team
SES: Socio-economic status
UTOPIAN: University of Toronto Practice Based Research Network
