# Supplementary figures and images for: Implementation of targeted screening for poverty in a large primary care team in Toronto, Canada: a feasibility study
Source: BMC Fam Pract. 2021 Sep 30;22:194. doi: 10.1186/s12875-021-01514-9 (PMC8483428; doi:10.1186/s12875-021-01514-9)

Additional file 1: Logic model


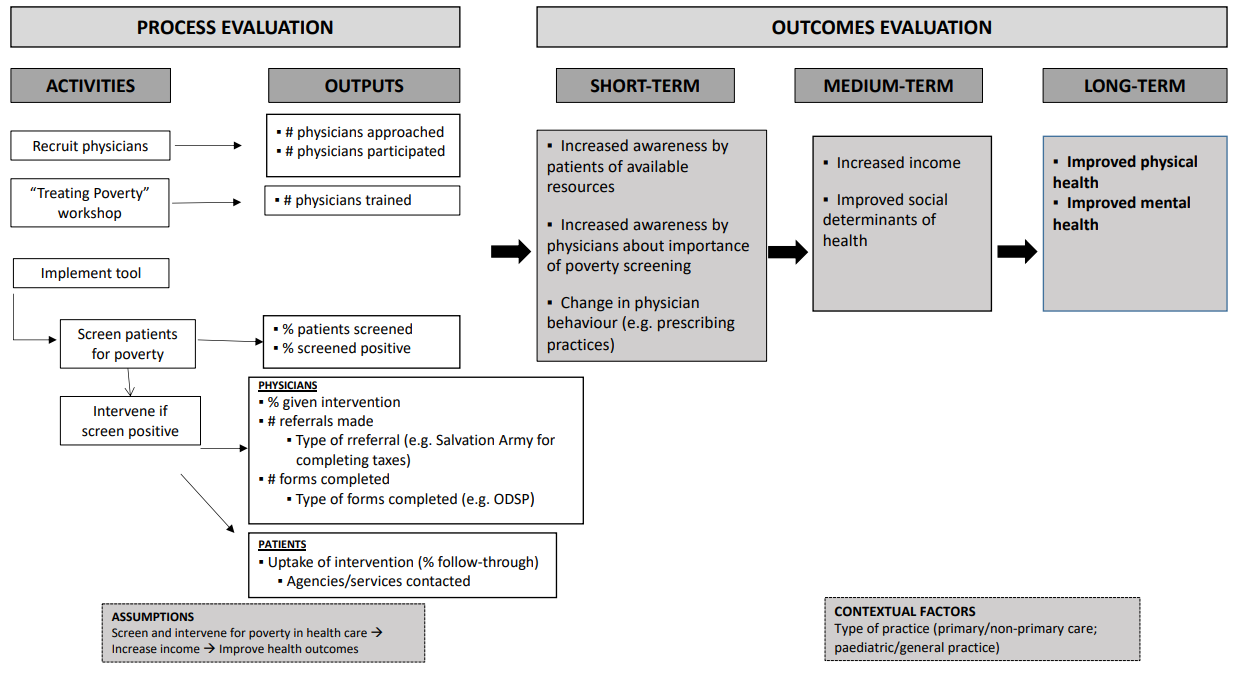

Supplement: Supplementary file 1 — Additional file 1. Logic model. [file 12875_2021_1514_MOESM1_ESM.docx]

Additional File 2: Standardized template for poverty screening and workflow


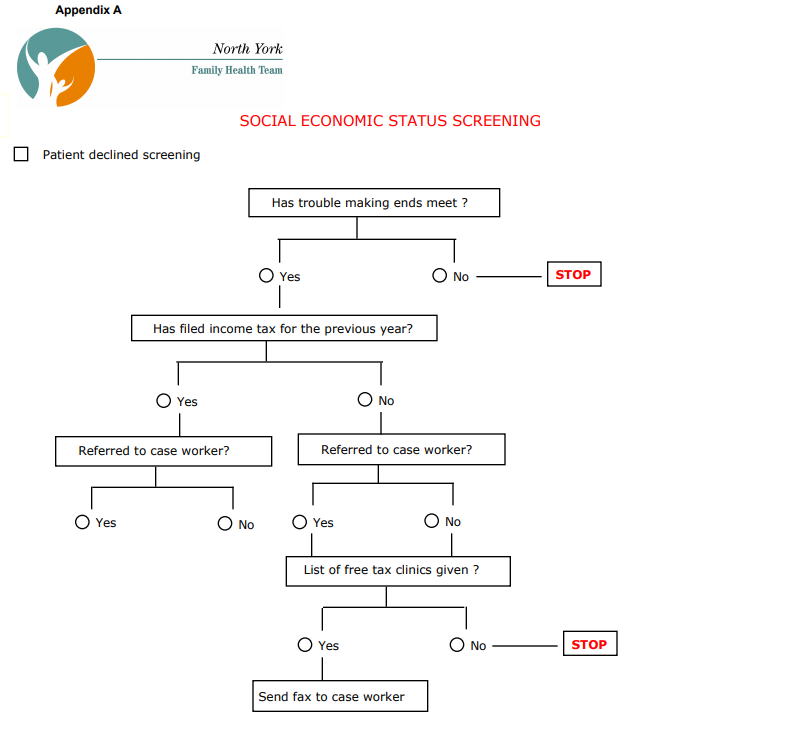

Supplement: Supplementary file 2 — Additional file 2. Standardized template for poverty screening and workflow. [file 12875_2021_1514_MOESM2_ESM.docx]
